# Infection against infection: parasite antagonism against parasites, viruses and bacteria

**DOI:** 10.1186/s40249-019-0560-6

**Published:** 2019-06-15

**Authors:** Shi-Shi Shen, Xiao-Yan Qu, Wei-Zhe Zhang, Jian Li, Zhi-Yue Lv

**Affiliations:** 1 Zhongshan School of Medicine, Sun Yat-sen University, Guangzhou, 510080 China; 2 Fifth Affiliated Hospital, Zhongshan School of Medicine, Sun Yat-sen University, ZhuHai, Guangdong China; 3 Key Laboratory of Tropical Disease Control, Ministry of Education, Sun Yat-sen University, Guangzhou, China; 4 Provincial Engineering Technology Research Center for Biological Vector Control, Guangzhou, China

**Keywords:** Antagonism, Parasite, Coinfection, Pathogen, Immunomodulation

## Abstract

**Background:**

Infectious diseases encompass a large spectrum of diseases that threaten human health, and coinfection is of particular importance because pathogen species can interact within the host. Currently, the antagonistic relationship between different pathogens during concurrent coinfections is defined as one in which one pathogen either manages to inhibit the invasion, development and reproduction of the other pathogen or biologically modulates the vector density. In this review, we provide an overview of the phenomenon and mechanisms of antagonism of coinfecting pathogens involving parasites.

**Main body:**

This review summarizes the antagonistic interaction between parasites and parasites, parasites and viruses, and parasites and bacteria. At present, relatively clear mechanisms explaining polyparasitism include apparent competition, exploitation competition, interference competition, biological control of intermediate hosts or vectors and suppressive effect on transmission. In particular, immunomodulation, including the suppression of dendritic cell (DC) responses, activation of basophils and mononuclear macrophages and adjuvant effects of the complement system, is described in detail.

**Conclusions:**

In this review, we summarize antagonistic concurrent infections involving parasites and provide a functional framework for in-depth studies of the underlying mechanisms of coinfection with different microorganisms, which will hasten the development of promising antimicrobial alternatives, such as novel antibacterial vaccines or biological methods of controlling infectious diseases, thus relieving the overwhelming burden of ever-increasing antimicrobial resistance.

**Electronic supplementary material:**

The online version of this article (10.1186/s40249-019-0560-6) contains supplementary material, which is available to authorized users.

## Multilingual abstracts

Please see Additional file 1 for translations of the abstract into the five official working languages of the United Nations.

## Background

Infectious diseases are imposing a considerable socioeconomic burden on countries with a wide distribution of pathogens. According to statistics from the World Health Organization (WHO), in 2016, lower respiratory infections ranked 3^rd^ among the top 10 causes of death, while diarrhoeal diseases caused by pathogenic infection ranked 8^th^, leading to an alarming death toll of millions of individuals worldwide [1]. More seriously, because of the rapid development of antimicrobial resistance (AMR) among pathogens, antibiotics are gradually losing their expected therapeutic capacity [2]. Therefore, the discovery of an alternative treatment strategy for controlling infections is imperative. Previous studies have revealed an interesting “infection against infection” relationship among various pathogens [3, 4]. In the antagonistic phenomenon, one pathogen from a certain species manages to suppress other species of pathogens during concurrent infections through either of the following two processes: biologically controlling pathogen vectors or inhibiting the invasion, development and reproduction of other pathogens. In this review, antagonistic relationships during coinfection that specifically involve parasites are summarized and discussed to motivate further research on the cusp of revealing or even utilizing the biological principles behind antagonism in tropical disease control. Taking *Wolbachia as* a quintessential example, this genus of gram-negative bacteria succeeds in limiting the transmission of parasites, such as malaria parasites and *Filaria* as well as emerging human arboviruses, when transferred into mosquitoes [5–11]. Additionally, certain nematodes and bacteria not only block the transmission of some trematodes by biologically controlling intermediate hosts but also relieve symptoms by suppressing infections by viruses and malaria parasites [12–14]. The “infection against infection” antagonism between parasites and parasites, parasites and viruses, or parasites and bacteria has been gradually applied to humans and has provided new insights into the development of novel effective antimicrobial therapies.

## Main text

### Literature Searching Strategy

**Fig. 1:**
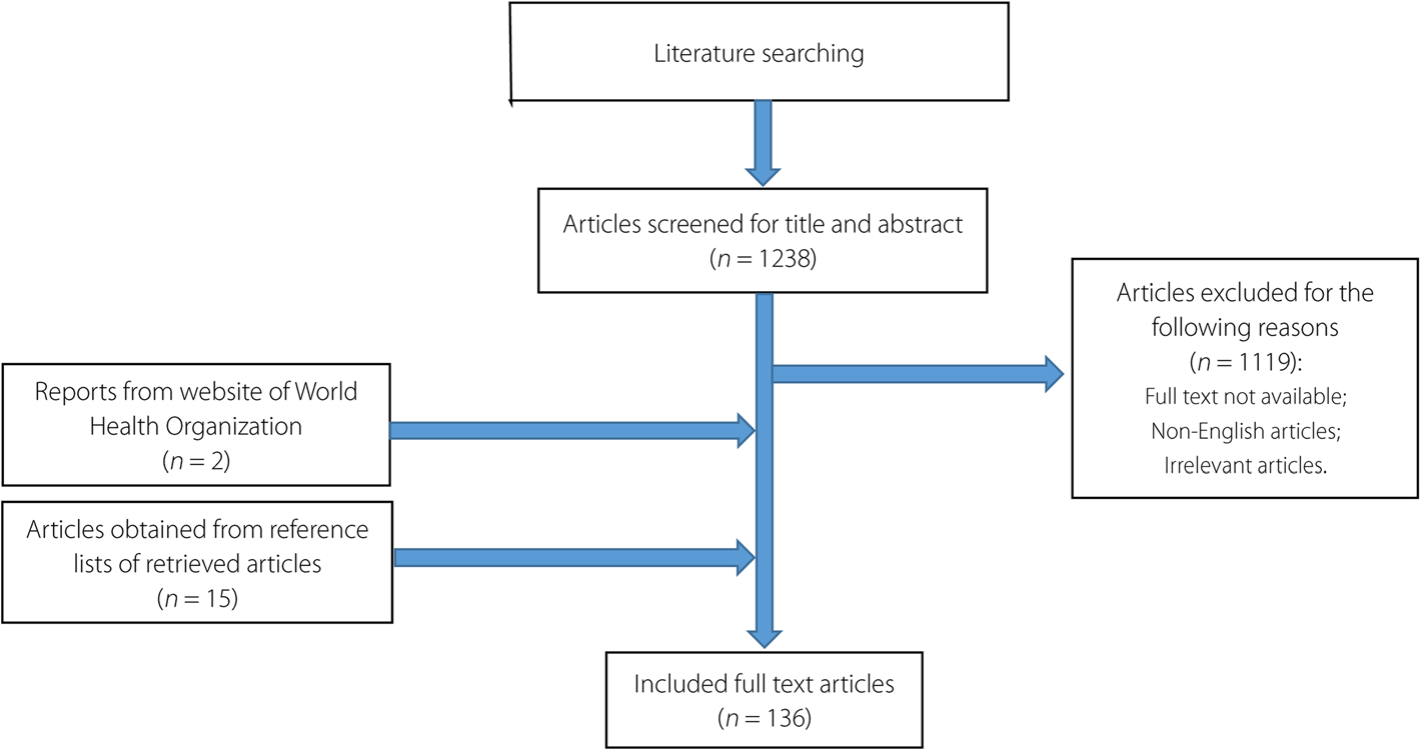
Flow chart of selection process of the included and excluded articles

Figure 1 presents the entire literature review process. In the present paper, we reviewed scientific studies published from 1970 to 2018 to identify studies focusing on antagonism among parasites and other pathogens during coinfection. A comprehensive search strategy was developed in PubMed, employing proper key words and free text terms. The search terms were “Coinfection” [Mesh] AND (“parasitology” [Subheading] OR “parasitology”[All Fields] OR “parasites” [All Fields] OR “parasites” [MeSH Terms]). Selected reference lists of retrieved articles were also searched manually using the online databases PubMed (https://www.ncbi.nlm.nih.gov/pubmed/) or Web of Science (http://apps.webofknowledge.com/UA_GeneralSearch_input.do?product=UA&search_mode=GeneralSearch&SID=5ChWtwlripdAqJRyqsq&preferencesSaved=). In addition, data about the incidence of diseases and antimicrobial resistance were obtained from the WHO website (http://www.who.int/). All identified articles were screened. The inclusion criteria were (1) studies that were published from Jan 1970 to Oct 2018; (2) studies that were published in English; and (3) studies that focused on antagonism between parasites and other pathogens. The exclusion criteria included (1) studies whose full articles were not available online either for free or with subscription (payment); (2) non-English literature; (3) studies irrelevant to the antagonism between parasites and other pathogens; and (4) poor-quality studies.

Two reviewers independently screened the PubMed electronic database to identify potentially eligible articles based on their titles and abstracts. All the research articles that were identified from searches of the electronic database were imported into a reference management software (EndNote X9, Thomson Corporation, Stanford, USA). Then, the study quality was independently assessed by two skilled researchers. The reference lists of the related articles were also checked to avoid missing relevant studies. The full text of each article was assessed carefully for inclusion or exclusion in the study when the information in the title and abstract was inadequate. Finally, we reviewed the identified studies to evaluate the eligibility on the basis of the inclusion and exclusion criteria. All the above procedures were conducted by two independent and trained researchers at Zhongshan School of Medicine in Sun Yat-sen University, and inconsistency between authors was resolved through discussions.

### Phenomena of infection against infection

Previous studies have shown the antagonistic interaction between parasites and parasites, parasites and viruses, and parasites and bacteria (Table 1). These “infection against infection” phenomena could possibly contribute to the development of a novel prevention strategy for infectious diseases.

**Table 1:** Phenomena of “infection against infection”

Categories	Interspecies antagonism	Patterns of antagonism	Intensity of antagonism	References
Between Parasites and parasites	*Heterorhabditis baujardi* against *Fasciola hepatica*^c^;	Inhibiting transmission	66.66%	[12]
	*H. baujardi* against *F. gigantica*^c^;	Inhibiting transmission	66.66%	[12]
	*Angiostrongylus cantonensis* against *Schistosoma mansoni*^c^;	Inhibiting transmission	12.45%	[13]
	*Trichinella spiralis* against *Plasmodium berghei*^c^;	Decreasing parasitaemia	47.50%	[15]
	*Ascaris lumbricoides* against *P. falciparum*^a,d^;	Attenuating symptoms	24.00%	[16]
	*Trichuris trichiura* against	Attenuating symptoms	27.50%	[17]
	*P. falciparum* ^d^	Decreasing parasitaemia	28.00%	[18, 19]
	*S. haematobium* against *P. falciparum*^a^	Attenuating symptoms	76.00%	[20]
	*S. mansoni* against *P. knowlesi*;	Inhibiting propagation	88.82%	[21]
	*S. mansoni* against *P. yoelii*^*a*^;	Inhibiting propagation	31.70%	[22]
	*Litomosoides sigmodontis* against *P. berghei*;	Inhibiting propagation	22.00%	[23]
	*Trichostrongylus colubriformis* against *Haemonchus contortus*;	Decreasing parasitaemia	74.07%	[24]
	*Babesia microti* against *P. cynomolgi*			
Between Parasites and bacteria	*Enterobacter amnigenus* against *P. vivax* ^*c*^;	Inhibiting transmission	94.37%	[25]
	*Enterobacter cloacae* against *P. vivax* ^*c*^;	Inhibiting transmission	83.10%	[25]
	*Serratia marcescens* against *P. vivax* ^*c*^;	Inhibiting transmission	98.59%	[25]
	*Ewingella americana* against *P. falciparum* ^*c*^;	Inhibiting transmission	38.00%	[26]
	*Salmonella typhimurium* against *Schistosoma*;	Decreasing parasitaemia and attenuating symptoms	56.82%,	[27]
	*Giardia muris* against *Citrobacter rodentium*;	Inhibiting propagation	42.86%	[28]
	*Wolbachia (wMelPop strain)* against *P. falciparum* ^*c*^;	Decreasing parasitaemia	46.78%	[29]
	*Wolbachia (wMelPop strain)* against *P. berghei* ^*c*^;	Decreasing parasitaemia	33.00%	[11]
	*Wolbachia (wMelPo strain)* against *P. gallinaceum* ^*c*^	Inhibiting propagation	79.50%	[30]
			80.00%	
Between Parasites and Viruses	*Plasmodium* against *chikungunya* virus*;*	Attenuating symptoms	39.50%	[31, 32]
	*Heligmosomoides polygyrus* against *respiratory syncytial* virus*;*	Attenuating symptoms	42.06%	[33]
	*Echinoparyphium* against *ranavirus*	Decreasing viral loads	23.50%	[34]

^a^Contradictory results

^b^Antagonism between different strains of *P. berghei*

^c^Laboratory conditions

^d^Conditions in human population

#### Parasites against parasites

Nematodes are widely distributed parasites that adapt successfully to nearly every ecosystem, and they are able to suppress the invasion, development and reproduction of certain parasites from other species. An animal study demonstrated decreased *Plasmodium berghei* parasitaemia in adult mice coinfected with *Trichinella spiralis.* This enhanced resistance to malaria was partially attributed to the nematode-induced activation of the mononuclear phagocyte system and a decrease in reticulocyte levels [15]. Additional evidence provided by another retrospective study indicated that malaria patients previously infected with helminths, such as *Ascaris lumbricoides*, *T. trichiura*, hookworm and *Strongyloides stercoralis*, were less likely to have mature schizonts in their peripheral blood or tissues or to suffer from renal failure, jaundice and severe cerebral malaria than those without protective helminth infections [16, 17, 35]. Moreover, two randomized trials identified a negative interaction between *A. lumbricoides* and *Plasmodium falciparum* [36, 37], while another trial in Nigeria showed a contrasting result [38]. Similar protection also occurred between filarial parasites and malarial parasites. To see this, consider an example involving BALB/c mice coinfected with *Litomosoides sigmodontis* and *P. berghei*. Mice did not develop blood-stage malaria [22], but their condition deteriorated unless filarial infection achieved patency [39]. Another example is the significant reduction in the intensity of malarial attack caused by the co-occurrence of *Brugia malayi* and *B. pahangi* infection [40]. Despite the protection of filarial parasites against *Plasmodium*, synergism was observed between filarial and other protozoa (*Wuchereria bancrofti* and malarial parasites, *B. malayi* and *Leishmania donovani*) [41, 42]. In addition, other studies confirmed that nematodes blocked the transmission of some trematodes by regulating the population of fresh water snails and intermediate hosts. For instance, after being transferred into snails, *Heterorhabditis baujardi* induced parasitic castration and consequently contributed to an average death rate of 67% in *Lymnaea columella,* the intermediate host of *Fasciola hepatica* and *Fasciola gigantica*. Therefore, *H. baujardi* was expected to become a feasible alternative for the prevention of fasciolosis [12]. Similarly, parasitic castration also occurred in *Biomphalaria glabrata* experimentally infected with *Angiostrongylus cantonensis* [13]. Although nematodes had the potential to suppress other parasitic infections, the interaction between coinfecting nematodes was intriguing and required further study. Pre-existing *Trichostrongylus colubriformis* negatively affected the survival of *Haemonchus contortus,* but contradictory results occurred with different infection orders [23]. The primary pathogen infecting the host was likely a determining factor in the relationship between *Tetracapsuloides bryosalmonae* and *Myxobolus cerebralis* [43]*,* but obvious antagonism was found between another two protozoa, *Babesia microti* and *Plasmodium cynomolgi* [24]*.* Interestingly, other studies indicated a positive association between concurrent protozoa in exacerbating clinical manifestation [44, 45] or increasing susceptibility of the hosts to the other protozoan, implying that in-depth studies are needed to clarify interactions between coinfecting protozoa [46]. In human populations, some studies have been conducted to generate an in-depth understanding of the interaction between concomitant infections with malaria parasites and *Schistosoma*, but these studies showed contradictory results. Some studies suggested a protective role for helminths [18–21, 47–50]; for example, one cross-sectional study demonstrated lower *P. falciparum* parasite densities in children with mild *S. haematobium* infection than those in the control group [18, 19], and a further study suggested an age-dependent manner of the protective effect [47]. Animal experiments also reported similar results where parasite growth and gametocyte infectivity of *Plasmodium yoelii* or *Plasmodium knowlesi* were both inhibited by coinfection with *Schistosoma mansoni* [20, 21]*.* However, other studies claimed that concurrent exposure to *P. falciparum* and *S. mansoni* might be synergistically associated in elevating parasitaemia, [51–53] exacerbating clinical manifestations [54–56] and increasing infection risks [57–60].

Surprisingly, an antagonistic interaction was also identified between diverse strains of malaria parasites. The total parasite densities in mice infected with virulent strains of *P. berghei* were reduced if mildly infective strains were inoculated into the hosts in advance [61].

#### Parasites against viruses

Parasites were also found to reduce the severity of viral infections. *Giardia lamblia* might modulate the pathogenicity of rotaviruses if concurrent infections occur [62]. Compared with rotavirus monoinfections, *G. lamblia*-rotavirus coinfections triggered diarrhoeal episodes of reduced severity [62]. A similar protective effect was discovered between *Plasmodium* and chikungunya virus (CHIKV) [31, 32]. Moreover, in animal experiments, nematodes such as *T. spiralis* and *Nematospiroides dubius* showed the ability to alleviate pathological changes caused by influenza A, while *T. spiralis* could suppress inflammatory infiltration in the lungs and downregulate cytokine production in the bronchoalveolar lavage fluid, and *N. dubius* was demonstrated to reduce viral titres in the lungs [35, 63]. Similar protection was discovered in *Heligmosomoides polygyrus*-infected mice, which demonstrated significantly attenuated pulmonary diseases after respiratory syncytial virus (RSV) [33]. In addition to protozoa and nematodes, trematodes (*Echinoparyphium*) were also negatively associated with viruses (ranavirus) by decreasing virus loads [34]. Conversely, other helminths were synergistically connected with some viruses by accelerating disease progression (lymphocytic choriomeningitis virus and *Leishmania guyanensis*, Toscana virus and *L. guyanensis*, Friend virus *and L. sigmodontis,* hepatitis C virus and *S. mansoni*) [64–66] or in increasing virus loads [67]. However, the relationship between human immunodeficiency virus (HIV) and parasites remains unclear. Some studies demonstrated that viral replication was diminished under *Trypanosoma cruzi* infection, while more evidence suggested that coinfection with parasites promotes viral replication [68], cell-to-cell transmission of virus [69] and exacerbation of clinical manifestations [70, 71].

#### Parasites against bacteria

Malaria is a mosquito-borne infectious disease that has captured the attention of international health organizations. According to the World Malaria Report, in 2017, 216 million cases were reported in 91 countries, and the global death toll reached 445 000. The elimination of malaria has been urgently advocated by the WHO [2]. The key to eliminating malaria is blocking the transmission of malaria parasites via the malarial vector, namely, the *Anopheles* mosquito. The life cycle of malaria parasites might be inhibited by many bacteria, for example, enterobacteria. When *Enterobacter amnigenus*, *Enterobacter cloacae* and *Serratia marcescens* were transferred along with *P. vivax*-infected blood into *Anopheles albimanus*, the infection rate of the coinfected mosquitoes decreased significantly compared with that of the control group; notably, the mean oocyst level in mosquitoes coinfected with *E. cloacae* was 2.5 times lower than that of the control group [25]. Moreover, coinfection with *S. marcescens* resulted in a decrease in the mosquito mortality rate [25]. By the same token, the formation of *P. falciparum* oocysts in *A. stephensi* was reported to be hindered by coinfection with gram-negative bacteria, such as *Escherichia coli, Pseudomonas aeruginosa* and *Ewingella americana* [26]*.* Another study showed a reduction in the number of adult schistosomal worms and eggs and a relief of schistosomiasis symptoms during coinfection with *Salmonella typhimurium* [27]. In regard to coinfection with *P. yoelii*, the susceptibility of hosts to *S. typhimurium* increased [72]. In contrast, the growth of enterobacteria (*Citrobacter rodentium*) was suppressed by coinfection with *Giardia muris* or *Giardia duodenalis* [28].

In addition to enterobacteria, *Wolbachia pipientis*, bacteria mainly infecting arthropods and nematodes, performed an essential function in malaria control. Somatic infections of *Anopheles gambiae* with the wMelPop strain of *Wolbachia* led to decreased oocyst levels and parasitic densities of *P. falciparum* [29] and *P. berghei* [11]. Furthermore, the wAlbB strain of *Wolbachia* induced *P. falciparum* resistance in *Anopheles stephensi,* a major vector of malaria parasites in South Asia and the Middle East [10]. Similar interactions were observed between *Wolbachia* (wMelPop strain)-infected *Aedes aegypti* and *P. gallinaceum* [8]*.* Interestingly, the blocking of malaria parasites by *Wolbachia* was modulated by temperature [30]. A model system of *P. yoelii* and *A. stephensi* revealed a reduction in both parasitic prevalence and oocyst intensity at 28 °C, unchanged parasitic prevalence and increased oocyst intensity at 24 °C, and unchanged parasitic prevalence and unchanged oocyst intensity at 20 °C [30].

*Wolbachia* played vital roles in vector modulation for the control of filariasis in tropical and subtropical areas. The infection of *A. aegypti* with *Wolbachia* prior to infection with *B. pahangi* microfilariae induced striking reductions in both the prevalence of infected mosquitoes and the mean number of infective parasites [9]. Nevertheless, additional evidence is needed to demonstrate the potential ability of *Wolbachia* to treat filariasis in humans in consideration of *B. pahangi*’s role as a filarial nematode in rodents. In general, studies on *Wolbachia* have provided exciting prospects for the elimination of mosquito-borne diseases, such as malaria and dengue.

Interactions between *Mycobacterium tuberculosis* and other concurrent pathogens were also highly studied. With current studies, we can temporarily conclude a synergistic relationship between *M. tuberculosis* and helminths in disease progression [73–75].

In conclusion, the antagonistic association between parasites and other concurrent pathogens was manifested as reduced susceptibility of hosts, growth suppression, and decreased transmission or attenuated clinical manifestations. The intriguing findings of antagonism between helminths and other various pathogens increase the comprehensive understanding of the mutualistic relationships between different pathogens. Elucidation of the mechanisms behind these antagonistic interactions is necessary; this knowledge will be conducive to controlling infectious diseases with a higher efficiency.

### Mechanism of parasite antagonism against parasites, viruses and bacteria

#### Apparent competition: Immunomodulation

Coinfection is a common occurrence that predominantly alters disease progression. Due to the shared endemicity of pathogens in vast areas, coinfection between pathogens, helminths in particular, has been expected and feared [4, 76, 77]. Despite the limited number of available studies, the preliminary conclusion that interactions between helminths and other pathogens seem to be complex and bidirectional can be safely drawn [78–80]. Currently, studies suggest that helminth infection could lead to robust cytokine production that could promote the occurrence of malaria [19, 47, 48, 78, 79, 81]. Nevertheless, there is clinical and epidemiologic evidence to support the notion that helminth infection favours protection by reducing the density of pathogens and mitigating immunopathological injury [81]. Studies have been conducted in models of experimental cerebral malaria (ECM) to explore the underlying immunological mechanisms [14, 79]. The outcomes not only rested on the different genetic backgrounds of the hosts but also depended on the species and densities of the pathogens [82].

A bimodal immune response is the distinctive characteristics of malaria containing a Th1-type response for control of the initial parasitaemia and further Th2-mediated cytokine production for parasite clearance [4, 83]. Helminth infections, known as potent inducers of the Th2-type response, can downregulate the effects of a secondary Th1-dependent parasitic challenge [84–87].In the absence of the initial inflammatory stage, T cell-mediated antigen clearance would be blocked, and the rapid proliferation of pathogens would ensue. Additionally, uncontrollable inflammatory reactions could induce serious immunopathological lesions. Supplementary studies have confirmed that the robust Th2-type response induced by chronic helminth infection could suppress the proinflammatory Th1-type response [88–90]. The emphasis of discussion in this section revolves mainly around *Schistosoma,* such as *S. mansoni* [91–93], *S. japonicum* [94, 95] and *S. haematobium* [18, 96, 97]. In the context of a low burden of malaria parasites along with an increase in *Schistosome* cercariae, the protective antimalarial immune response would be enhanced [84]. Several potential mechanisms might induce the negative interaction between these players [98, 99]. However, no significant interaction between malaria parasites and *Schistosome* was observed as a result of challenge with a heavy malaria parasite burden during coinfection, and its causes remain unclear [84].

#### Suppression of dendritic cell (DC) responses to competitors

DC subgroups in the spleen mainly include plasmacytoid DCs (pDCs) and myeloid DCs (mDCs). Numerous studies have confirmed that DCs are able to induce the adaptive immune response in the initial phase of malaria parasite infection, during which the predominant Th1 immune response induces the development of cerebral malaria. Mature DCs are critical antigen-presenting cells (APCs) that activate the Th1-type response and cytotoxic T cell response. In the early stage of malaria, phagocytosis by DCs is enhanced to selectively identify and remove pRBCs. The activation of naive T cells and the polarization switch from Th0 to Th1 are closely related to the mature DC phenotype, which is marked by the upregulation of major histocompatibility complex (MHC) class II molecules and the CD80/CD86 costimulatory molecules. The high-level expression of MHC class II molecules expedites the processing and presentation of malaria parasite antigens by DCs to activate CD4+ T cells and hence trigger the Th1 and cytotoxic T lymphocyte responses. The activation of CD8+ T cells by malaria parasite infection may further jeopardize the microvascular endothelial tissue and blood-brain barrier (BBB) through the activity of the perforin/granzyme pathway [100] and the LT-α pathway [101], which in turn allows malaria parasite components and other potential damage factors to enter the brain parenchyma. Consequently, the overactivation of glial cells is induced, and the apoptosis of astrocytes becomes uncontrolled. During monoinfection of malaria pathogens, a striking increase in the vascular permeability of the brain tissue was observed [94]. However, during challenges by relatively low densities of malaria parasites in the coinfection, increased helminth loads contributed to an apparent decrease in the counts of mature DCs along with a further decrease in functional DC responses to induce a Th1 predisposition, thereby providing crucial protection against ECM pathogenesis [19, 37, 47, 94, 102].

Exposure to helminths not only dampens the maturation of DCs but also promotes the population expansion of regulatory T (Treg) cells. Immature DCs can produce IL-10 and/or TGF-β in response to exposure to helminth excretory-secretory products. In vitro experiments demonstrated that IL-10 and TGF-β were essential cytokines that induced the differentiation and amplification of Treg cells from naive T cell precursors and promoted the further expansion of pre-existing Treg cell subpopulations [61]. The dynamic balance between proinflammatory and anti-inflammatory responses is indispensable for maintaining homeostasis. After the proinflammatory Th1-type response is established in the early stage of parasitic infection, specific antibodies produced by plasma cells with the help of Th2 cells could efficaciously remove worms and prevent disease recurrence [94]. Treg cells, such as CD4+CD25+Foxp3+, are capable of maintaining proper immune homeostasis by regulating the production of proinflammatory and anti-inflammatory cytokines and by mediating the differentiation of naive T cells into diverse subgroups, such as Th1, Th2 and Treg cells [47, 88].

#### Activation of basophils and mononuclear macrophages against competitors

During the occurrence and progression of cerebral malaria, the deposition of pRBCs in cerebral capillaries causes the adhesion of immune cells and platelets to vascular endothelial cells, thus resulting in encephalorrhagia, hydrocephalus and BBB permeability changes. Among them, the infiltration of T cells (especially CD8+ T cells) is critical for the increase in the permeability of the BBB and brain tissue injury. The excessive release of proinflammatory cytokines, chemokines and adhesion molecules is closely related to the above process, especially IFN-γ.

With the demonstration of a positive correlation between the burden of schistosomal infection and Th2 cytokine synthesis, helminth-induced activation of basophils and mononuclear macrophages may be the critical mechanism underlying the Th2-type response bias and alleviation of BBB impairment [103].

In response to schistosomal antigens, such as IPSE/α-1, a major secretory glycoprotein antigen from eggs of *Schistosome*, the notable upregulation of Th2 cytokines was detected, IL-4 and IL-10 [104] in particular, suggesting that in the Treg-mediated anti-inflammatory response, IL-4 and IL-10 might predominate in inhibiting the Th1-type response in malaria [94, 105], and IL-4 could suppress the IFN-γ-secreting Treg cells. The Th1-biased differentiation of T cells into IFN-γ-producing effectors was markedly down-modulated in response to IL-4 treatment [103]. The reduction in IFN- γ caused the production of chemokines (such as CXCL9 and CXCL10) and adhesion molecules (such as ICAM-1), the sequestration and deposition of parasite-infected red blood cells (pRBCs), leukocytes and platelets, and the migration of CD8+ T cells into the brain to be restrained [105]. Furthermore, IL-4 could activate GATA3, the specific transcription factors of Th2 cells, by signalling pathways (e.g., STAT6 pathway, transcription factors, even self-regulation). Conversely, GATA3 promoted the positive regulation of IL-4 production in Th2 cells and led to a positive-feedback loop. In accordance with the previous situation, significant changes were not observed in severe malarial infections [91]. With the exception of basophils and monocytes, antigens alternatively activated M2 macrophages, which then secreted IL-10 and other anti-inflammatory cytokines. IL-10, in contrast to IL-4, might target Th1 effector functions by preventing the production of IFN-γ[106]. Likewise, Th2 cells release the relevant cytokines to drive M2 polarization of macrophages to form the positive-feedback effect [107]. Regarding *Schistosoma* infection, the bulk of the reported data from ECM models indicated a decrease in the expression level of mRNAs to be translated into proinflammatory factors associated with brain histopathological characteristics. Furthermore, alterations in the parasite density could result in corresponding alterations in the above parameters [91]. Alleviation of microvascular lesion formation and BBB permeability provided noteworthy improvements in symptom severity by reducing the activation of CD8+ T cells in the brain and alleviating neurotoxicity [95].

Consistent with the results of a series of previous studies, the immune environment of chronic helminth infection appears to favour the induction of counter-regulatory responses suppressing infection by other pathogens [83]. Several reports have revealed that helminth infection is closely involved in the decrease in viral burden in peripheral and placental blood [108, 109].

#### Adjuvant effect of complement system

It has been previously confirmed that the C3d component of the complement system acts as a bridge between innate and acquired immunity. The adjuvant effect of C3d has been exemplified in *S. mansoni* models [96]. It was found that C3d, a breakdown product of complement C3, was present in high amounts. C3d, containing the ligand for CD21, a B cell co-receptor, would be expected to bind to CR2 (CD21) to promote the development and maintenance of memory B lymphocytes. Different hypotheses have been developed to explain this function, including the tyrosine-phosphorylated association of CD21 with CD19 and the CD21 expression of follicular dendritic cells [96, 110, 111].

Under the influence of C3d, the complement system could select antigens for recognition by the acquired immune system and be complementary to some other components of innate immunity to notably raise the capability of anti-infection in hosts.

The above contents have been thoroughly studied at present. In addition, Toll-like receptors (TLRs) have been clarified to participate in antimalarial defence. While TLR9 responds to malaria pigments, TLR4 responds to glycosylphosphatidylinositol (GPI) produced by *P. falciparum*. However, the idiographic mechanism of action underlying these responses has not been elucidated. TLR-mediated immunopathological damage of brain tissue is linked to the aggregation of CD8+ T cells, DCs, and natural killer (NK) cells. Experimental research has already disclosed the downregulated expression of TLRs in the presence of *Schistosoma* cercariae [96]. The specific mechanism still needs further exploration.

Coincidentally, antibody-mediated immune modulation also occurred in the interactions between some pathogens. Higher levels of *Clonorchis sinensis*-specific IgG and IgA elicited upon *T. spiralis* infection have been the subject of several reports, indicating that enhanced *C. sinensis* clearance induced by coinfection was associated with systemic and mucosal IgG and IgA responses [112, 113].

#### Exploitation competition: Resource limitation

If two or more organisms have similar needs for limited resources and space, fierce competition for survival opportunities and reproductive capacity enhancements will inevitably ensue [4, 114, 115]. Competition occurs at times in a vast geographic distribution, and the environment of coinfection is no exception.

If the struggle for existence among these organisms persists long enough, adaptive evolution might occur in order for each organism to occupy a favourable position. This process, termed exploitation competition, is divided into two strategies: divergent resource use between the organisms when suitable alternative resources are available and adaptations that improve the ability to acquire those shared resources when there are no alternatives [116, 117]. Taking advantage of exploitation competition is devastating to competitors. Tissue tropism is believed to explain the phenomenon that some species of trematodes prefer to infect a particular host tissue. Despite evidence indicating the tropism of *Diplostomum hupehensis* to the intestine of the avian host and to different eye tissues of the fish host [116], the direct demonstration of the underlying mechanism remains unknown thus far. However, the latter exploitation competition strategy is more applicable, especially in regions of high malaria endemicity [114]. The mutation of enzymes could drive the conversion of *Plasmodium* stages to favour the asexual stages over the single sexually differentiated stages, thus simultaneously supporting both the reduced transmission efficiency and the increased infection efficiency. A rosetting phenotype in which uninfected cells adhere to a central infected cell has been observed. Pathogens might be expected to burst from the central infected cell and specifically target peripheral cells in rosettes, but this reasonable hypothesis has not yet been tested [118].

#### Interference competition: Direct attack or exclusion

In addition to the abovementioned mechanisms, coinfection might enter into interference competition by producing toxic compounds that directly eliminate or hinder the development of pathogenic competitors [61, 118, 119].

Epidemiological and experimental studies have revealed that intestinal symbiotic bacteria could stimulate the innate immune system of arthropods and upregulate the expression of multiple genes inhibiting other infections, such as cecropins, defensins and gambicin [28, 61, 118, 120]. Interference competition also occurred in certain cestodes, which could release substances termed “crowding factors” [116]. Crowding factors are arguably the least ambiguous interference strategy: they seem to be explained as a passive accumulation of end metabolites serving as a signal for worms to slow their growth; however, crowding factors have adaptive value in moderating the proliferation of competitors when resources are limited. Regretfully, research on crowding factors was tortuous, progressed slowly and has gradually been ignored in recent years [21, 121]. Hence, clarification of this uncertainty remains elusive.

##### References to biological control of disease transmission in intermediate hosts or vectors

Abundant epidemiological investigations and experimental studies suggested that several nematode species could interrupt the expansion of infectious diseases by reducing or even obliterating the reproductive capacity of intermediate hosts or vectors. This suggestion appeared to be borne out by recent research on entomopathogenic nematodes (EPNs) and symbiotic bacteria. EPNs carry symbiotic bacteria, which, after the EPNs enter the host, are released in the intestinal cavity [122] and produce a fatal neurotoxic metabolite. The nematodes then reproduce in the dead host, and the new generation of offspring invades the next host [123]. The role of EPNs in snails needs further exploration. The biological control of intermediate hosts or vectors is likely to confer considerable prophylactic and therapeutic benefits [124].

#### Proteolysis and inversion in the excretion pattern

Numerous experimental results noted a significant decrease in the concentration of total protein in infected snails, which occurred gradually as the infection advanced. A case in point is the infection of *B. glabrata* by *A. cantonensis*. The microbiological interaction involved was accompanied by a substantial increase in the level of urea and a significant reduction in the concentration of uric acid in the haemolymph derived from infected specimens [125], suggesting that nematode infections induce proteolysis and an inversion in the excretion pattern of infected snails. According to the related enzymatic investigation, the activity levels of alanine aminotransferase (ALT) and aspartate aminotransferase (AST) were markedly higher in the infected groups than in the control group. These changes produced an increasing rate of amino acid deamination, thus supplying alternative substrates for gluconeogenesis. Moreover, the accumulation of urea accelerated the urea cycle; thus, more arginine was available for parasite development [121]. Interestingly, the nitrogen degradation products could produce noxious effects on the host neuroendocrine system and even kill the infected snails, thus creating a comfortable environment for nematodes to accomplish larval-stage development. The reduction in the population of intermediate hosts carrying pathogens has a positive impact on inhibiting the transmission of infectious diseases, especially in natural epidemic foci (e.g., the transmission of angiostrongyliasis in Guangdong, China) [122, 126].

#### Degenerative capacity

Current research has demonstrated that nematodes weaken the reproductive capacity of snails mainly by direct impairment of the reproductive tissues and organs or by indirect harm through the withdrawal of nutrients [12, 13]. The former mechanism suggested that nematodes induced egg granuloma formation and fibrosis, rendering the snail reproductive organs vulnerable. Over time, the inflammatory infiltration and fibrosis within cephalopods hindered motor function, thus affecting the feeding and fertilization abilities of snails. The latter mechanism speculated that nematodes competed with the host to obtain essential nutrients for proliferation, the outcome of which was the suppression of the reproductive capacity of the snails. The galactogen synthesized by the albumen gland, for instance, served as the primary energy source for snail embryos and newly hatched offspring. A galactogen deficiency in the albumen gland would decelerate the hatching rate of infected snails, thus characterizing parasitic castration as a nutritional process [12].

#### Suppressive effect on transmission

*Wolbachia* is a renowned intracellular endosymbiont of invertebrates that is capable of protecting insects from pathogens and limiting their ability to transmit mosquito-borne pathogens by the reproductive manipulation of their hosts by means such as sperm-egg cytoplasmic incompatibility (CI), which results in the production of unviable progeny when a male mosquito carrying *Wolbachia* mates with a wild female mosquito [127, 128]. The above interaction was reported to occur during the successful introduction of *Wolbachia* into the mosquito species (e.g., *Aedes*, *Anopheles* and *Culex*), which are the major vectors of human pathogens, including protozoa (*Plasmodium* sp.), filariae and a variety of viruses (causing dengue, yellow fever, and West Nile) [129]. Specialists were inspired to apply this mechanism of antagonism in the venues for malaria control.

The development of various pathogens in mosquitoes was confirmed to be inhibited by immune preactivation, thus suggesting that constitutive immunoregulation could influence the transmission of infectious pathogens to humans [124]. A more in-depth study conducted recently showed the capability of *Wolbachia* to induce immune system upregulation. *Wolbachia* stimulated mosquitoes to produce reactive oxygen species (ROS) in testes and ovaries; this ROS production later activated the Toll pathway and resulted in the synthesis of a variety of antibacterial peptides, such as defensins and cecropins, thus suppressing other coinfected pathogens [9, 130].

Another striking example of this surprising ability of *Wolbachia* is that the over-replicating wMelPop strain of *Wolbachia* could induce the constitutive expression of innate immune genes in mosquitoes [9], thus implying a potential explanation for the interspecific competitive relationships between different parasites as a contributory factor to the life-shortening parasitic phenotypes [106, 131].

## Conclusions

Previous studies have illuminated the antagonism between parasites and parasites, parasites and viruses, and parasites and bacteria (Table 1). Further research has described four possible mechanisms underlying this phenomenon. In particular, the immunomodulation theory, which indicates that coinfection was able to reduce the immunopathological alterations caused by the Th1-type immune response, has been widely accepted [4, 91]. A low intensity of *Schistosoma* infection could improve the protective antimalarial immune response associated with the expression of related cytokines [18, 91, 94, 132], the permeability of the BBB [94], the DC response [94], and the differentiation and activation of Tregs [94], thereby potentially being protective against cerebral malaria. Vector modulation is another universally acknowledged hypothesis to explain antagonistic coinfection; quintessential examples of vector modulation include the effects of EPNs and *Wolbachia* [12, 13, 106, 123, 125, 127, 128, 133]. Other mechanisms underlying antagonism include interference competition and exploitation competition [61, 116, 134].

In summary, antagonistic phenomena between coinfecting pathogens are common, but persistent efforts are required to elucidate the underlying mechanisms and identify more effective strategies for combating pathogenic infection [77, 135]. Though antagonism involving parasites has been highlighted in this review, the unexpected “infection against infection” relationship has also been observed in the cotransmission of bacteria and viruses. One of the most promising examples is that the *Wolbachia* strains that had been successfully established in wild *Ae. Aegypti* to control dengue virus are now undergoing field trials in dengue endemic areas of Australia, Brazil, Indonesia and Vietnam [136]. The “infection against infection” relationship will offer novel control strategies for infectious diseases, such as biological control of parasitic vectors and potent vaccine development.

## Additional file

Additional file 1: Multilingual abstracts in the five official working languages of the United Nations. (PDF 345 kb)

## Data Availability

The datasets used and/or analyzed during the current study are available from the corresponding author upon reasonable request.

## Abbreviations

ALTA: lanine aminotransferase
AMR: Antimicrobial resistance
APC: Antigen-presenting cell
AST: Aspartate aminotransferase
BBB: Blood-brain barrier
CI: Cytoplasmic incompatibility
DC: Dendritic cell
ECM: Experimental cerebral malaria
EPN: Entomopathogenic nematode
GPI: Glycosylphosphatidylinositol
mDC: Myeloid dendritic cell
MHC: Major histocompatibility complex
NK: Natural killer cell
pDC: Plasmacytoid dendritic cell
pRBC: Parasite-infected red blood cell
ROS: reactive oxygen species
TLR: Toll-like receptor
Treg: Regulatory T cell
WHO: World Health Organization
