# Supplementary material for: Infection against infection: parasite antagonism against parasites, viruses and bacteria
Source: Infect Dis Poverty. 2019 Jun 15;8:49. doi: 10.1186/s40249-019-0560-6 (PMC6570864; doi:10.1186/s40249-019-0560-6)
Supplement: Supplementary file 1 — Multilingual abstracts in the five official working languages of the United Nations. (PDF 345 kb) [file 40249_2019_560_MOESM1_ESM.pdf]

Translation of the abstract into the five official working languages of the United Nations

العدوى ضد العدوى: المناهضة الطفيلية ضد الطفيليات، الفيروسات والبكتيريا

شي شي شين ، شياو يان تشو ، وى زه تشانغ ، وجيان لي ، وزهي يو

نبذة مختصرة

معلومات عامة: تشمل الأمراض المعدية مجموعة كبيرة من الأمراض التي تهدد صحة الإنسان ، وتكتسي العدوى المرافقة أهمية خاصة لأن الأنواع المسببة للأمراض يمكنها التفاعل داخل المضيف. في الوقت الحاضر، تُعرَّف العلاقة المعادية بين مسببات الأمراض المختلفة أثناء العدوى المرافقة و المتزامنة على أنها علاقة يستطيع فيها أحد مسببات الأمراض من منع غزو، تطور وتكاثر مسبب الأمراض الآخر أو أن يعدل بيولوجيا كثافة الناقل . في هذه المراجعة، نقدم نظرة عامة عن ظاهرة وآليات العداء الخاصة بمسببات الأمراض التي تشتمل على طفيليات.

المتن الرئيسي: تلخص هذه المراجعة العلاقة العدائية بين الطفيليات و الطفيليات، الطفيليات و الفيروسات و الطفيليات والبكتيريا. في الوقت الحاضر، تتضمن الآليات الواضحة نسبيًا الموضحةللتطفل المتعدد المنافسة الظاهرية، منافسة الاستغلال، منافسة التداخل، التحكم البيولوجي للمضيفات المتوسطة أو المتجهات و التأثير القمعي على الإنتقال على وجه الخصوص، التعديل المناعي ، بما في ذلك قمع استجابات الخلايا الجذعية (DC) ، وتنشيط الخلايا القاعدية والبلاعم أحادية النواة والتأثيرات المساعدة للنظام المكمل موصوفة بالتفصيل.

الاستنتاجات: في هذه المراجعة ، نلخص االعدوى المتزامنة المعادية التي تنطوي على الطفيليات ونوفر إطارًا وظيفيًا للدراسات المعمقة للآليات الأساسية للعدوى المرافِقة مع الأحياء الدقيقة المختلفة، والتي سوف تسرع في تطوير بدائل واعدة مضادة للميكروبات ، مثل لقاحات مضادة للجراثيم جديدة أو طرق بيولوجية للسيطرة على الأمراض المعدية ، وبالتالي تخفيف العبء الساحق لأي مقاومة متزايدة مضادة للميكروبات.

Translated from English version into Arabic by Irene Campo, Revised by Aberzak Mohamed, through

TRANSLATORS
WITHOUT BORDERS

以感染控制感染：寄生虫与寄生虫、病毒、细菌间的拮抗

沈施施，屈晓妍，张炜哲，李坚，吕志跃

摘要

引言：世界范围内，感染性疾病正对人类健康构成严重威胁。而不同种类的病原体在宿主体内发生相互作用从而引起的共感染现象，有望为控制感染性疾病的传播与流行提供行之有效的新策略。不同种类病原体在共感染发生时的相互拮抗关系，指的是其中一种病原体能够抑制另一种病原体在共同宿主体内的侵袭与增殖过程，或对传播另一种病原体的载体密度进行相应生物学调控。本篇综述旨在对共感染的不同病原体之间的拮抗现象与拮抗机制做出归纳总结，并对其在感染性疾病治疗与控制当中的运用前景进行综合评估。

正文：本综述概述了寄生虫与寄生虫、病毒、细菌之间的共感染拮抗作用，介绍了包括表观竞争、资源利用性竞争、干扰竞争在内的作用模式，以及参与共感染的其中一种病原体通过发挥对中间宿主或载体的抑制作用来阻断另一病原体传播的生物学效应。此外，本文还详细

阐述了树突状细胞免疫应答受抑、嗜碱性粒细胞和单核巨噬细胞的激活、补体系统辅助效应等不同病原体共感染现象背后的免疫调节机制。
结论：本综述总结并讨论了与寄生虫有关的共感染过程中不同病原体之间的拮抗作用、相应机制及广阔运用前景。了解不同病原体在同一宿主体内的共感染现象的研究进展，将有助于藉由研发全新抗感染疫苗或利用其它生物学手段对感染性疾病进行有效防控。

Translated from English version into Chinese by 吕志跃、沈施施、屈晓妍、张炜哲

Infection contre infection : antagonisme parasitaire contre les parasites, virus et bactéries.

Shi-Shi Shen, Xiao-Yan Qu, Wei-Zhe Zhang, Jian Li et Zhi-Yue Lv

Résumé
Contexte : Les maladies infectieuses couvrent un large éventail de pathologies qui menacent la santé humaine. La notion de co-infection revêt une importance particulière car les espèces pathogènes interagissent dans l'organisme hôte. On définit actuellement la relation antagoniste entre les différents pathogènes participant à une co-infection comme une relation dans laquelle un agent pathogène parvient à inhiber l'invasion, le développement et la reproduction de l'autre ou module biologiquement la densité de ses vecteurs. Nous présentons ici un tour d'horizon du phénomène et des mécanismes d'antagonismes entre les pathogènes, y compris les parasites, qui participent ensemble à une co-infection.
Discussion : La présente revue résume les interactions antagoniques entre parasites et parasites, entre parasites et virus et entre parasites et bactéries. On peut aujourd'hui expliquer le polyparasitisme par des mécanismes relativement clairs : concurrence apparente, concurrence d'exploitation, concurrence d'interférence, contrôle biologique des hôtes intermédiaires ou des vecteurs et effet de suppression de la transmission. L'immunomodulation, en particulier, est décrite en détail. Elle inclut la suppression des réponses des cellules dendritiques, l'activation des basophiles et des macrophages mononucléaires et des effets adjuvants sur le système du complément.
Conclusions : Nous décrivons ici des infections concurrentes antagonistes par des parasites et posons le cadre fonctionnel d'études approfondies des mécanismes qui sous-tendent la co-infection par différents micro-organismes. Ces travaux accéléreront le développement de nouveaux antimicrobiens prometteurs, tels que de nouveaux vaccins antibactériens ou de nouvelles méthodes biologiques de lutte contre les maladies infectieuses, afin d'alléger le fardeau de plus en plus écrasant de la résistance aux antibiotiques.

Translated from English version into French by Suzanne Assenat, Revised by Perrine Corre, through

TRANSLATORS
WITHOUT BORDERS

Инфекция против инфекции: Антагонизм среди паразитов, вирусов и бактерий

Ши-Ши Шэнь, Сяо-Янь Цюй, Вэй-Чжэ Чжан, Цзянь Ли и Чжи-Юэ Лу

Резюме

Обоснование: К инфекционным болезням относится широкий спектр заболеваний, представляющих опасность для здоровья людей. Особую угрозу представляет коинфекция, поскольку различные виды возбудителей могут взаимодействовать внутри хозяина. На сегодняшний день антагонизмом различных микроорганизмов при коинфекциях называют взаимодействие, при котором одному патогену удается либо ингибировать инвазию, развитие и репродукцию другого возбудителя либо биологически влиять на плотность переносчиков. В данном обзоре мы рассматриваем феномен и механизм антагонизма микроорганизмов при паразитарных коинфекциях.

Основной текст: В данном обзоре рассматривается антагонизм между паразитами, паразитов с вирусами и паразитов с бактериями. В настоящий момент относительно понятные механизмы, объясняющие полипаразитизм включают в себя борьбу за выживание, борьбу за использование ресурсов, недопущение использования ресурсов другим видом паразитов, биологический контроль промежуточного хозяина или переносчиков и подавление путей передачи. В частности, подробно описана иммуномодуляция, включающая подавление восприимчивости дендритных клеток (ДК), активацию базофилов и мононуклеарных макрофагов, а также адъювантное действие системы комплемента.

Заключение: В данном обзоре мы рассматриваем антагонистические конкурентные паразитарные инфекции и предлагаем функциональную концепцию углубленных исследований основополагающих механизмов коинфекций различными микроорганизмами, которые ускорят разработку альтернативных противомикробных препаратов, таких как новейшие антибактериальные вакцины или биологические методы контроля инфекционных болезней, что облегчает бремя постоянно возрастающей резистентности микробов к антибиотикам.

Translated from English version into Russian by Nataliya Zhydkikh, Revised by Alexander Somin, through

TRANSLATORS
WITHOUT BORDERS

Infección contra infección: antagonismo parásito contra parásitos, virus y bacterias

Shi-Shi Shen, Xiao-Yan Qu, Wei-Zhe Zhang, Jian Li y Zhi-Yue Lv

Resumen

Antecedentes: Las enfermedades infecciosas abarcan un gran espectro de enfermedades que amenazan la salud humana; además, la coinfección es de particular importancia porque las especies

patógenas pueden interactuar con el huésped. En la actualidad, la relación antagonista entre diferentes patógenos durante coinfecciones simultáneas se define como una en la cual un patógeno o logra inhibir la invasión, el desarrollo y la reproducción del otro patógeno o modula de manera biológica la densidad del portador. En este análisis, proveemos un resumen del fenómeno y los mecanismos del antagonismo de los patógenos coinfecciosos relacionados con parásitos.
Texto principal: Este resumen sintetiza la interacción antagonista entre parásitos y parásitos, parásitos y virus, y parásitos y bacterias. En la actualidad, los mecanismos relativamente claros que explican el poliparasitismo incluyen la competencia aparente, la competencia por explotación, la competencia por interferencia, el control biológico de los huéspedes o portadores intermediarios y el efecto supresor en la transmisión. Se describen, con especial detalle, la inmunomodulación, incluidos la supresión de las respuestas de las células dendríticas (CD), la activación de basófilos y macrófagos mononucleares, y los efectos adyuvantes del sistema complementario.
Conclusiones: En este resumen, sintetizamos las infecciones antagonistas concomitantes relacionadas con parásitos y proveemos un marco de trabajo funcional para estudios detallados sobre los mecanismos subyacentes de la coinfección con diferentes microorganismos; lo cual apresurará el desarrollo de alternativas antimicrobianas prometedoras, tales como novedosas vacunas antibacterianas o métodos biológicos para el control de enfermedades infecciosas, y por consiguiente, mitigará la enorme carga de la creciente resistencia antimicrobiana.

Translated from English version into Spanish by Ilduara Escobedo, Revised by Maria Paula Gorgone, through

TRANSLATORS
WITHOUT BORDERS
